# Response to the *Correspondence* of Helbing et al. *“Mouse sepsis models: don't forget ambient temperature!”*

**DOI:** 10.1186/s40635-022-00451-w

**Published:** 2022-05-23

**Authors:** Michael Bauer, Reinhard Wetzker

**Affiliations:** Department of Anesthesiology and Intensive Care Medicine, Jena University Hospital, 07747 Jena, Germany

We are grateful for these comments on this important aspect. Helbing et al. [1] convincingly translate our generalized view on the importance of abiotic stress factors in sepsis on the ambient temperature of sepsis models in the research context. Indeed, considering thermoneutral—or in a broader sense “stressless”—conditions appears as a key prerequisite for productive elaboration of host responses to infection.

In our opinion, neglecting the impact of biotic and abiotic stressors represents a main cause for conflicting results in sepsis research. Hence, the study mentioned by Helbing et al. revealing increased mortality of LPS-treated mice at housing temperature below thermoneutrality [2] sharply contrasts to another recent report using the same sepsis model for investigations of the energetic trade-off between LPS-induced immune responses and thermoneutrality [3]. In conflict with the former investigations, Ganeshan et al. observed significant reduction of the mortality of LPS-treated mice at ambient temperature below thermoneutral. In addition, these authors show that another stressor, food restriction, is producing the same vitality promoting effect [3].

How to interpret these contradictory results? One obvious explanation might be the different LPS doses applied in various studies. Whereas Ndongson-Dongmo et al. [2] used 10 mg LPS/kg body mass, Ganeshan et al. [3] applied only 3 mg LPS/kg maximum. Reduced LPS challenge seems to turn the mice from “cold-sensitive” to a “cold-tolerating” phenotype. To rationalize this enigma, we may hint to the hormesis theory outlined in our review. Low doses of the given stressor LPS express ability to increase vitality (and resilience to cold stress) and high doses provoke damage in the affected organism.

Sepsis patient studies incidentally also uncover contrasting results about the effects of ambient temperature on the morbidity and mortality of critical illness. In this context, the controversy regarding targeted temperature management in patients after cardiac arrest—an ultimately severe stress event—might also shed light on the problem: whereas initial reports [4–6] revealed promising impact of targeted hypothermia, larger multi-center clinical trials failed to confirm these observations [7, 8]. We do not share the generalized conclusion of Helbing et al. that “Clinical data clearly indicate that spontaneous lowering of Tc is correlated with a poor outcome of sepsis.”, but might emphasize the possibility of missing patient stratification in the multi-center clinical trials. Whereas Schortgen et al. [6] disclosed beneficial effects of external cooling on a specific cohort of febrile patients in septic shock, the multi-center studies [7, 8] comprised a broad spectrum of patients with sepsis symptoms. It is well known that sepsis patients include at least two phenotypes, one exhibiting hyperinflammation and another one with hypothermia [9]. As a conclusion of the patient study by Schortgen et al. [6] and on the basis of animal experiments [3], one might predict that preferentially hyperinflammatory febrile patients in overt shock and oxygen debt make profit from external cooling.

In sum, we share the opinion of Helbing et al. [1] to include thermoneutral/stressless conditions in experimental investigation of host responses to infectious attacks. In this vein, the ecological approach in experimental sepsis research together with a strict patient stratification may pave the way for significant progress in translational efforts for novel ideas to treat this fatal disease.
